# Ten-Year Outcomes of Patients with Left Main Coronary Artery Disease and Diabetes Mellitus Treated with Percutaneous Coronary Intervention

**DOI:** 10.3390/jcm14248851

**Published:** 2025-12-14

**Authors:** Jola Bresha, Gjin Ndrepepa, Constantin Kuna, Thorsten Kessler, Isabella Hintz, Paul Justenhoven, Tareq Ibrahim, Sebastian Kufner, Heribert Schunkert, Marco Valgimigli, Gert Richardt, Karl-Ludwig Laugwitz, Salvatore Cassese, Adnan Kastrati, Jens Wiebe

**Affiliations:** 1 Medizinische. Klinik, Klinikum Rechts der Isar, Universitätsklinikum der Technischen Universität München, 80636 Munich, Germany; jola.bresha@mri.tum.de (J.B.); tareq.ibrahim@mri.tum.de (T.I.); kl.laugwitz@mri.tum.de (K.-L.L.); 2 Deutsches Herzzentrum München, Department of Cardiology, Universitätsklinikum der Technischen Universität München, Lazarettstr. 36, 80636 Munich, Germany; kuna@dhm.mhn.de (C.K.); thorsten.kessler@tum.de (T.K.); hintz-hintz@dhm.mhn.de (I.H.); justenhoven-justenhoven@dhm.mhn.de (P.J.); sebastian.kufner@tum.de (S.K.); cassese@dhm.mhn.de (S.C.); kastrati@dhm.mhn.de (A.K.); jens.wiebe@googlemail.com (J.W.); 3 DZHK (German Centre for Cardiovascular Research), Partner Site Munich Heart Alliance, 80636 Munich, Germany; 4 Division of Cardiology, Cardiocentro Ticino Institute, Ente Ospedaliero Cantonale, 6900 Lugano, Switzerland; marco.valgimigli@eoc.ch; 5 Department of Cardiology, Klinik Bad Odelsloe, 23843 Bad Odelsloe, Germany; g.richardt@asklepios.com

**Keywords:** diabetes mellitus, left main coronary artery disease, mortality, percutaneous coronary intervention

## Abstract

**Background/Objectives:** Long-term outcomes of patients with left main coronary artery (LMCA) disease and diabetes mellitus (DM) undergoing percutaneous coronary intervention (PCI) are incompletely investigated. The aim of this study was to assess the 10-year clinical outcomes after PCI according to diabetic status and antidiabetic therapy in patients with LMCA. **Methods:** This study represents a pooled analysis of two randomized trials (*n* = 1257 patients) on LMCA PCI focused on the prespecified subgroups of diabetic patients. Patients were categorized in groups according to the diabetic status and antidiabetic therapy (oral drugs or insulin therapy). The primary endpoint was 10-year all-cause mortality. **Results:** Overall, 361 patients had DM (246 patients on oral antidiabetic drugs and 115 patients on insulin therapy) and 896 patients had no DM. At 10 years, 477 patients died: 291 nondiabetic patients (35.7%), 111 diabetic patients (49.5%) on oral antidiabetic drugs and 75 diabetic patients (70.0%) on insulin therapy (hazard ratio [HR] = 1.57, 95% confidence interval [1.26–1.96]; *p* < 0.001 for diabetic patients on oral antidiabetic drugs vs. nondiabetic patients; HR = 2.80 [2.17–3.61]; *p* < 0.001 for diabetic patients on insulin therapy vs. nondiabetic patients; HR = 1.78 [1.33–2.39]; *p* <0.001 for diabetic patients on insulin therapy vs. diabetic patients on oral antidiabetic drugs). The 10-year incidence of myocardial infarction was higher in diabetic patients on insulin therapy (10.0%) versus diabetic patients on oral antidiabetic drugs (3.0%). There were no significant differences between the groups regarding the 10-year incidence of definite stent thrombosis, coronary artery bypass graft surgery, repeat PCI or stroke. **Conclusions:** In patients with LMCA disease undergoing PCI, DM was associated with a higher 10-year incidence of all-cause mortality than patients without DM with the worst outcomes observed in diabetic patients on insulin therapy.

## 1. Introduction

Critical atherosclerotic lesions in the left main coronary artery (LMCA) are found in 3–5% of patients undergoing coronary angiography and 10–30% of patients undergoing coronary artery bypass graft surgery (CABG) [1]. Diabetes mellitus (DM) is an established cardiovascular risk factor that is associated with more severe and diffuse coronary artery disease (CAD), increased platelet reactivity and propensity to vascular thrombosis, higher systemic inflammation, reduced survival and poorer outcomes following percutaneous coronary intervention (PCI) or CABG [2,3,4,5,6]. Approximately 25% of patients with LMCA disease have DM, which confers a higher long-term (5-year) risk of death, spontaneous myocardial infarction, stroke and repeat revascularization compared with patients without DM [5]. Historically, CABG has been considered as a preferred revascularization strategy (as compared to PCI) in patients with LMCA disease. Recent advances in the field of coronary interventions have led to a 4-fold rise in PCI procedures for LMCA disease and more favorable subsequent outcomes [7]. A recent meta-analysis of randomized trials that compared PCI with CABG in patients with LMCA disease gave mixed results with no difference between CABG and PCI with respect to 5-year death and stroke and a higher risk of myocardial infarction and revascularization with PCI, albeit without diabetes-by-revascularization strategy interaction [8]. However, studies that have investigated the efficacy of PCI versus CABG in patients with LMCA disease and DM have offered controversial results [9,10]. Since, patients with DM represent a heterogeneous group and the risk conferred by LMCA disease and DM may be additive in case both conditions co-exist, a careful decision-making and a personalized approach in selecting the most appropriate revascularization strategy in these patients are required [11,12]. We undertook this study to assess the 10-year clinical outcomes after PCI in patients with LMCA and DM and to investigate whether clinical outcomes differ according to the type of antidiabetic therapy.

## 2. Methods

### 2.1. Patients

This study represents a pooled analysis of 2 randomized trials on LMCA PCI focused on the prespecified subgroups of diabetic patients: the Drug-Eluting-Stents for Unprotected Left Main Stem Disease (ISAR-LEFT-MAIN) trial (NCT00133237; *n* = 607 patients, 176 patients with DM) [13] and the Intracoronary Stenting and Angiographic Results: Drug-Eluting Stents for Unprotected Coronary Left Main Lesions (ISAR-LEFT MAIN-2) trial (NCT00598637; *n* = 650 patients, 185 patients with DM) [14]. Overall, 1257 patients >18 years of age with ischemic symptoms or documented myocardial ischemia in the presence of ≥50% de novo stenosis located in the LMCA were included in this analysis. Patients presenting with ST-segment elevation myocardial infarction within <48 h from the chest pain onset or those with previous CABG, in-stent restenosis, cardiogenic shock, acute infections, malignancies or other comorbidities with a life expectancy of less than one year, planned elective surgery requiring discontinuation of P2Y_12_ antithrombotic drugs in the first 6 months following enrollment (in source studies), reported allergy to the study drugs or stent constituents, pregnancy or previous enrollment in the trials were excluded. Patients obtained from the ISAR-LEFT MAIN trial received a paclitaxel-eluting stent or a sirolimus-eluting stent whereas patients obtained from the ISAR-LEFT MAIN 2 trial, received a zotarolimus-eluting stent or an everolimus-eluting stent [13,14]. Written informed consent and institutional ethics committee approval (code: 1339/05; date 28 June 2005 and code: 1975/07; date 18 December 2007) were obtained in all patients. The study was conducted in accordance with the Declaration of Helsinki.

### 2.2. Study Definitions

All included patients had a ≥50% de novo stenosis located in the LMCA, which encompassed a coronary artery segment from the left main stem ostium to the end of the 5 mm proximal segments of the left anterior descending artery, left circumflex artery and ramus intermedius if the latter had a vessel size of ≥2 mm in diameter. LMCA stenosis was classified as ostial (located within 3 mm of the LMCA ostium), mid-shaft (located in the medial part of LMCA with at least 3 mm of apparently nondiseased artery before bifurcation) or distal (located in the distal part of the LMCA and bifurcation/trifurcation with the proximal left anterior descending artery, proximal left circumflex artery and proximal ramus intermedius if this vessel was present). The stenting technique was left to the discretion of the operators. Coronary angiograms were digitally recorded and analyzed off-line in the quantitative angiographic core laboratory with an automated edge-detection system (CMS version 7.1, Medis Medical Imaging Systems, Leiden, The Netherlands) by independent, experienced operators who were blinded to the treatment allocation. The SYNTAX (SYNergy between PCI with TAXUS and Cardiac Surgery) was defined according to Sianos et al. [15]. Details are provided in the primary studies [13,14]. DM was diagnosed if the patient had an established diagnosis of the disease and was under active treatment with insulin or oral hypoglycemic drugs. For patients with a de novo diagnosis of DM during the index hospitalization, DM was diagnosed according to the World Health Organization criteria: an abnormal fasting blood glucose (≥126 mg/dL or ≥7.0 mmol/L) or abnormal glucose tolerance test (≥200 mg/dL or ≥11.1 mmol/L). Measurements of glucated hemoglobin HbA1c were used to assess stress-induced hyperglycemia. Glycated hemoglobin A1c (HbA1c) was measured with a turbidimetric inhibition immunoassay method in hemolyzed whole blood anticoagulated with tripotassium-EDTA. Serum creatinine was measured with a kinetic colorimetric assay based on the compensated Jaffe method. Left ventricular ejection fraction was calculated using the area-length method on left ventricular angiograms. Body mass index was calculated using the patient’s weight and height measured during the hospital stay. Other cardiovascular risk factors—arterial hypertension, hypercholesterolemia and smoking were defined as per guideline-recommended criteria at the time of enrollment of patients in the source studies.

Drug therapy at hospital discharge included clopidogrel 75 mg/day or prasugrel 10 mg/day for at least 12 months and aspirin at a dose of 80 to 200 mg daily given indefinitely. Other medications were prescribed at the discretion of the treating physician.

### 2.3. Study Endpoints and Follow-Up

The primary endpoint of the study was all-cause mortality at 10 years. Cardiac mortality, myocardial infarction, definite stent thrombosis, target lesion revascularization, nontarget lesion revascularization and stroke at 10 years were also analyzed. Cardiac death and definite stent thrombosis were defined according to the Academic Research Consortium criteria [16]. The diagnosis of myocardial infarction was established in the presence of new Q waves on the electrocardiogram and/or documentation of elevated creatine kinase-MB isoform (or creatine kinase) to at least two times the upper limit of reference range in at least two blood samples. Target lesion revascularization was defined as any repeat PCI involving the left main area or CABG surgery in at least one of the main left coronary vessels due to luminal renarrowing in association with symptoms or objective signs of ischemia. Nontarget lesion revascularization was defined as any repeat PCI not involving the LMCA region or CABG of the nontarget vessel due to pre-existing disease, disease progression or other reasons not related to the target lesion. Stroke was defined as an acute neurological event lasting 24 h or longer with focal signs and symptoms and without evidence supporting an alternative explanation. The diagnosis of stroke was confirmed by brain imaging tests or pathological examination.

The follow-up included phone interviews at one month, six months and one year and yearly thereafter up to 10 years. Information on deaths was obtained from the hospital records, death certificates and phone contact with the patient’s relatives or referring physician, the insurance companies and registration-of-address office. The follow-up data were collected and adjudicated by personnel unaware of the clinical data of the patients.

### 2.4. Statistical Analysis

Continuous data are presented as median with 25–75th percentiles or mean ± standard deviation and compared with the Kruskal-Wallis rank sum test or ANOVA, when appropriate. The normality of distribution of continuous data was tested using the Kolmogorov-Smirnov test. Categorical data are shown as counts and proportions (%) and compared with the chi-squared test. The 10-year deaths are shown as cumulative incidences calculated with the Kaplan-Meier method. Other analyzed outcomes are shown as cumulative incidences after accounting for the competing risk of death. Comparison of outcomes in groups according to diabetic status was performed with the univariable Cox proportional hazards model. The association between diabetic status and the study outcomes was adjusted for potential confounders using the multivariable Cox proportional hazards model. The level of multicolinearity among the variables was assessed by calculating the variance inflation factor (VIF). The following variables had a VIF < 5: age, sex, body mass index, arterial hypertension, hypercholesterolemia, current smoking, extent of CAD, clinical presentation (acute coronary syndrome or stable CAD), history of myocardial infarction, history of PCI, serum creatinine, left ventricular ejection fraction, comorbidities, vessel size, SYNTAX score, type of drug-eluting stent, occluded right coronary artery, coronary artery dominance, lesion location, trifurcation morphology and stenting technique. These variables were entered into the Cox proportional hazards model. The proportionality of hazards assumption was tested according to the Grambsch and Therneau method [17]. The statistical analysis was performed using the R 4.1.0 Statistical Software (The R Foundation for Statistical Computing, Vienna, Austria). A two-sided *p* < 0.05 was considered as statistically significant.

## 3. Results

### 3.1. Baseline Data

The study included 1257 patients who underwent PCI for unprotected LMCA disease. Of them 361 patients had DM. Baseline and angiographic/procedural data of patients with and without DM are shown in Supplemental Table S1 and Supplemental Table S2, respectively. Patients were categorized in groups according to the DM status and type of therapy: a group without DM (*n* = 896), a group with DM on oral antidiabetic drugs (*n* = 246) and a group with DM on insulin therapy (*n* = 115). Baseline data are shown in Table 1. There were significant differences between the groups with respect to the body mass index, proportions of patients with arterial hypertension, extent of CAD, serum creatinine, left ventricular ejection fraction, frequency of peripheral arterial disease and chronic obstructive pulmonary disease and the SYNTAX score. Among patients with diabetes, those on insulin therapy had significantly higher levels of glucated hemoglobin A1c than patients on oral antidiabetic drugs. The other variables did not differ significantly between the groups. Angiographic and procedural data are shown in Table 2. There were no significant differences across the groups except for the frequency of use of final kissing balloon. Main cardiovascular drugs prescribed at discharge are shown in Supplemental Table S3.

### 3.2. Clinical Outcome

**Figure 1:**
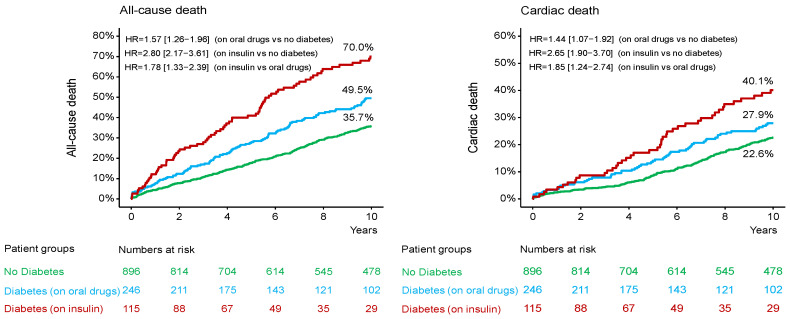
Kaplan-Meier curves of 10-year all-cause and cardiac mortality. HR = hazard ratio.

**Figure 2:**
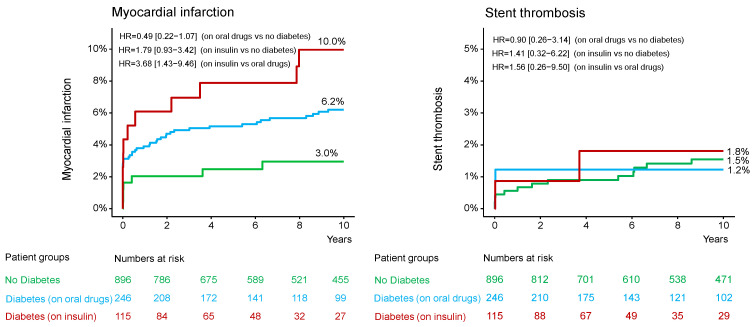
Kaplan-Meier curves of 10-year myocardial infarction and definite stent thrombosis. HR = hazard ratio.

In patients without DM, diabetic patients on oral antidiabetic drug and diabetic patients on insulin therapy, the median [25–75th percentiles] follow-up was 11.3 [10.0–13.5] years, 11.2 [7.8–13.6] years and 11.1 [4.3–12.1] years, respectively (*p* = 0.392). The 10-year clinical outcome according to diabetic status is shown in the Supplemental Table S4. The 10-year clinical outcome in the study groups is shown in Table 3. Deaths of any cause (the primary endpoint) occurred in 477 patients (38%): 291 patients (35.7%) without DM, 111 diabetic patients (49.5%) on oral antidiabetic drugs and 75 diabetic patients (70.0%) on insulin therapy (hazard ratio [HR] = 1.57, 95% confidence interval [1.26–1.96] for the group with DM on oral antidiabetic drugs vs. the group without DM; HR = 2.80 [2.17–3.61] for the group with DM on insulin therapy vs. the group without DM and HR = 1.78 [1.33–2.39] for the group with DM on insulin therapy vs. the group with DM on oral antidiabetic drugs; Table 3 and Figure 1). Cardiac deaths occurred in 285 patients (60% of deaths): 181 patients (22.6%) without DM, 62 diabetic patients (27.9%) on oral antidiabetic drugs and 42 diabetic patients (40.1%) on insulin therapy (HR = 1.44 [1.07–1.92] for the group with DM on oral antidiabetic drugs vs. the group without DM; HR = 2.65 [1.90–3.70] for the group with DM on insulin therapy vs. the group without DM and HR = 1.85 [1.24–2.74] for the group with DM on insulin therapy vs. the group with DM on oral antidiabetic drugs; Table 3 and Figure 1). The 10-year incidence of myocardial infarction was significantly higher in diabetic patients on insulin therapy compared with diabetic patients on oral antidiabetic drugs (Figure 2; Table 3). There were no significant differences between the groups with respect to the 10-year incidence of definite stent thrombosis (Figure 2) and CABG, repeat PCI or stroke (Table 3). Time-to-event curves of the occurrence of CABG or repeat PCI at 10 years are shown in Supplemental Figure S1. The number, type and location of coronary interventions are shown in Supplemental Table S5. The frequency of coronary interventions in each coronary artery, the time interval to the first repeat PCI and the time intervals between repeat PCIs are shown in Supplemental Table S6.

The 10-year clinical outcome according to metabolic control (assessed by hemoglobin A1c level) was analyzed in patients with DM. The median [95% confidence interval] value of hemoglobin A1c was 6.9% [6.3–7.7%]. Patients were categorized in groups according to the hemoglobin A1c < median (*n* = 146) and ≥median (*n* = 146). Clinical outcome according to the glucated hemoglobin level is shown in Supplemental Table S7.

The association between diabetic status, antidiabetic therapy and clinical outcome was adjusted in the multivariable Cox proportional hazards model (see Methods for variables entered into the model). The risk for 10-year all-cause mortality was higher in diabetic patients requiring insulin therapy than patients without DM (adjusted HR = 2.10 [1.58–2.78], *p* < 0.001) or diabetic patients on oral antidiabetic drugs (adjusted HR = 1.71 [1.25–2.32], *p* < 0.001). The risk for all-cause mortality was higher in diabetic patients on oral antithrombotic therapy than patients without DM but the level of statistical significance was not achieved (adjusted HR = 1.23 [0.98–1.55], *p* = 0.080). The same pattern of association was observed for cardiac mortality. The adjusted risk for myocardial infarction was significantly higher in diabetic patients on insulin therapy than diabetic patients on oral antidiabetic drugs (adjusted HR = 3.03 [1.09–8.43], *p* = 0.034). There were no significant associations between diabetic status and/or therapy and the risk for definite stent thrombosis, CABG, repeat PCI, stroke or nontarget lesion revascularization (Table 4).

## 4. Discussion

The main findings of the study may be summarized as follows: (1) In patients with LMCA disease undergoing PCI, DM was associated with a significantly higher incidence of 10-year mortality. (2) Patients with DM receiving insulin therapy had significantly higher rates of 10-year all-cause and cardiac mortality compared with patients without DM or diabetic patients on oral antidiabetic drugs. (3) Diabetic patients receiving insulin therapy had a significantly higher 10-year risk of myocardial infarction than diabetic patients on antidiabetic drugs. (4) There were no significant differences according to diabetic status or type of therapy among diabetic patients with respect to the 10-year rates of definite stent thrombosis, repeat PCI, CABG, target lesion revascularization, stroke or nontarget lesion revascularization.

Previous studies have demonstrated worse outcomes in diabetic patients with LMCA disease after PCI or CABG compared with nondiabetic patients. A pooled analysis of 4 randomized trials of patients with LMCA disease and a low-to-intermediate SYNTAX score who underwent PCI or CABG showed that DM was associated with higher 5-year rates of death, myocardial infarction, stroke or repeat revascularization [5]. The 5-year survival did not differ between PCI or CABG regardless of diabetic status [5]. Our study showed that an association between DM and higher risk of all-cause death after PCI for unprotected LMCA disease persists up to 10 years after intervention. However, the crucial finding of our study was the significant association between the type of antidiabetic therapy and 10-year mortality after PCI. The time-to-event curves showed a progressive widening of the difference in mortality between the groups with various types of antidiabetic therapy suggesting that the difference in the risk for mortality according to the type of antidiabetic therapy even may increase over time. The study by Gaba et al. [5]. also demonstrated gradients in the risk for all-cause and cardiovascular mortality across diabetic patients on insulin therapy (highest risk), diabetic patients on oral antidiabetic drugs and patients without DM (lowest risk). Other studies have also reported an association between the type of therapy in diabetic patients and the risk for mortality after PCI. The FAST-MI (French registry of Acute ST elevation or non-ST-elevation Myocardial Infarction) registry that included 1221 diabetic patients with acute myocardial infarction discharged alive from the hospital showed that insulin prescription at discharge was associated with 72% higher adjusted risk for mortality at 5 years [18]. A recent study that included 869 patients undergoing PCI for unprotected left main CAD showed that insulin-treated patients but not those treated with oral antidiabetic drugs had significantly higher rates of all-cause death, spontaneous myocardial infarction or major adverse cardiac and cerebrovascular events at one year [19]. Our study also showed that insulin-treated diabetic patients have an increased risk of mortality up to 10 years compared with patients without DM or diabetic patients on oral antidiabetic drugs. Even though in our study, diabetic patients on oral antidiabetic drugs had a higher risk of all-cause mortality compared with patients without DM, the association was attenuated after adjustment. However, the SYNTAX Extended Survival (SYNTAXES) study showed that both noninsulin and insulin therapy were independent correlates of 10-year mortality in patients with DM [20]. Nevertheless, the risk of mortality was higher in insulin-treated patients.

Our study adds to the existing evidence that insulin therapy signifies an increased risk for long-term mortality in diabetic patients with LMCA disease undergoing PCI. Although the underlying reasons for this finding are not entirely clear, a number of putative mechanisms may be offered. First, insulin therapy might have been initiated in diabetic patients in whom the adequate metabolic control was not achieved using the oral antidiabetic drug therapy. In this regard, insulin therapy may be seen as a marker of advanced and more severe disease, which is associated with a worse prognosis. Higher HbA1c values in patients receiving insulin therapy may denote difficulties in achieving an optimal metabolic control in these patients. Second, compensatory hyperinsulinemia is a hallmark of DM that may be further exacerbated by exogenous insulin [21]. Hyperinsulinemia including iatrogenic hyperinsulinemia, particularly if insulin is inadequately titrated, is associated with adverse events that increase the risk of cardiomyopathy, induce endothelial dysfunction, promote atherosclerosis, cause renal retention of sodium and water and increase vasoreactivity and blood pressure [21]. Cardiovascular effects of insulin therapy in diabetic patients have been recently reviewed [21]. Third, insulin therapy is associated with the risk of hypoglycemia [22,23]. Hypoglycemia, particularly if severe and recurrent, set into motion pathological events that markedly increase the risk for adverse outcomes, particularly in patients with high cardiovascular risk [21,24]. Hypoglycemia activates pro-inflammatory mediators [25,26], increases platelet and coagulation activation [27], increases oxidative stress [21], causes endothelial dysfunction and reduces systemic fibrinolysis [28]. These effects may promote a prothrombotic state that increases the risk of thrombotic events following PCI in diabetic patients. Hypoglycemia causes sympathetic nerve activation and catecholamine release increasing heart rate, systolic blood pressure, myocardial contractility and stroke volume leading to exacerbation of myocardial ischemia [29]. Thus, insulin-therapy-induced hypoglycemia may increase the risk of mortality in diabetic patients following PCI. The Action in Diabetes and Vascular Disease: Preterax and Diamicron Modified Release Controlled Evaluation (ADVANCE) study that included diabetic patients showed that severe hypoglycemia was associated with a higher adjusted risk for major macrovascular events, cardiovascular and all-cause mortality at 5 years [30].

The current study has strengths and limitations. The main strengths consist in including of a relatively large number of patients with LMCA disease, angiographic analysis performed in the centralized core laboratory, stringent criteria for event adjudication and long-term follow-up. The study has also a number of limitations. First, although the study patients were obtained from the randomized studies and the current analysis was prespecified in the setting of source trials, the antidiabetic therapy (and group definition) was prescribed on a nonrandomized basis. The patient categorization in groups according to the antidiabetic therapy was defined at discharge and we have no information with respect to any change in the antidiabetic therapy during the follow-up. Second, conditional on the time of recruitment of patients in the primary trials, the impact of newer generations of coronary stents, antithrombotic drugs and antidiabetic drugs (glucagon-like peptide 1 receptor agonists or sodium-glucose cotransporter 2 inhibitors) cannot be assessed. In addition, we have no information on the types (or dosis) of oral antidiabetic drugs prescribed or whether these drugs were used concomitantly with insulin in patients receiving insulin therapy. Third, the number of diabetic patients was relatively small. Moreover, in approximately 20% of patients with DM, the hemoglobin A1c values were missing. Thus, the limited number of events due to these factors may have led to an inconclusive analysis with respect to the association of metabolic control of DM with the clinical outcomes. Although we used the World Health Organization criteria for the de novo (during index hospitalization) diagnosis of DM, the impact of stress-induced hyperglycemia cannot be entirely neglected. Fourth, conditional on the time of patient enrollment in the source studies, patients included in this analysis (particularly patients presenting with acute coronary syndromes) may have received a somewhat outdated therapy in terms of drug-eluting stents and antithrombotic therapy [31]. Fifth, although we adjusted for a wide range of demographical and clinical variables, the potential impact of residual confounders on the association between diabetic status (and antidiabetic therapy) and clinical outcome cannot be ignored.

In conclusion, in patients with LMCA disease undergoing PCI, DM was associated with a higher 10-year incidence of all-cause mortality. Diabetic patients with LMCA disease on insulin therapy showed a higher risk of 10-year mortality compared with patients without DM or diabetic patients on oral antidiabetic drugs. Insulin therapy was an independent correlate of an increased risk of 10-year all-cause and cardiac mortality. By showing that patients with DM on insulin therapy did worse compared with other groups of patients, these findings may have implications with respect to the decision-making and a personalized approach in selecting the most appropriate revascularization strategy in diabetic patients with LMCA disease. Specifically designed and well-powered randomized studies are eagerly needed to establish the best management strategy in patients with LMCA disease and DM.

## Figures and Tables

**Table 1:** Baseline characteristics.

Characteristic	Without Diabetes (*n* = 896)	With Diabetes (*n* = 361)		*p* Value
		On Oral Antidiabetic Drugs (*n* = 246)	On Insulin Therapy (*n* = 115)	
Age (years)	69.4 [62.1; 76.2]	70.8 [65.5; 76.9]	70.4 [65.5; 77.1]	0.433
Women	206 (23.0%)	66 (26.8%)	29 (25.2%)	0.433
Body mass index (kg/m^2^)	26.2 [24.2; 28.7]	27.8 [25.2; 30.7]	28.0 [25.4; 31.6]	<0.001
Arterial hypertension	592 (66.1%)	193 (78.5%)	83 (72.2%)	0.001
Hypercholesterolemia	662 (73.9%)	183 (74.4%)	91 (79.1%)	0.478
Current smoking	113 (12.6%)	28 (11.4%)	11 (9.57%)	0.596
Extent of coronary artery disease				<0.001
2-vessel	287 (32.0%)	58 (23.6%)	14 (12.2%)	
3-vessel	609 (68.0%)	188 (76.4%)	101 (87.8%)	
Clinical presentation				0.051
Stable coronary artery disease	572 (63.8%)	146 (59.3%)	61 (53.0%)	
Acute coronary syndrome	324 (36.2%)	100 (40.7%)	54 (47.0%)	
History of myocardial infarction	257 (28.7%)	70 (28.5%)	31 (27.0%)	0.928
History of percutaneous coronary intervention	448 (50.0%)	125 (50.8%)	62 (53.9%)	0.728
Serum creatinine (mg/dl)	0.95 [0.80; 1.10]	1.00 [0.81; 1.20]	1.10 [0.90; 1.46]	<0.001
Glucated hemoglobin A1c (%)	-	6.67 [5.05; 13.1]	7.28 [5.32; 14.6]	<0.001
Left ventricular ejection fraction (%)	58.0 [47.8; 62.0]	55.5 [41.0; 60.0]	48.0 [35.0; 60.0]	<0.001
Comorbidities				
Peripheral artery disease	70 (7.8%)	38 (15.4%)	18 (15.7%)	<0.001
Chronic obstructive pulmonary disease	20 (2.2%)	14 (5.7%)	6 (5.2%)	0.012
Malignancies	151 (16.9%)	43 (17.5%)	15 (13.0%)	0.541
Vessel (left main) size (mm)	3.73 (0.51)	3.71 (0.52)	3.69 (0.50)	0.588
SYNTAX score	29.0 [21.0; 36.0]	30.0 [22.0; 38.0]	32.0 [24.5; 39.5]	0.014
Second generation drug-eluting stents	465 (51.9%)	124 (50.4%)	61 (53.0%)	0.877

Data are median with 25th; 75th percentiles or counts (%); SYNTAX = SYNergy between PCI with TAXUS and Cardiac Surgery.

**Table 2:** Angiographic and procedural characteristics.

Characteristic	Without Diabetes (*n* = 896)	With Diabetes (*n* = 361)		*p* Value
		On Oral Antidiabetic Drugs (*n* = 246)	On Insulin Therapy (*n* = 115)	
Lesion location in the left main coronary artery				0.240
Ostial	94 (10.5%)	31 (12.6%)	20 (17.4%)	
Distal	655 (73.1%)	172 (69.9%)	76 (66.1%)	
Mid-shaft	147 (16.4%)	43 (17.5%)	19 (16.5%)	
Occluded right coronary artery	103 (11.5%)	31 (12.6%)	19 (16.5%)	0.292
Trifurcation morphology	108 (12.1%)	31 (12.6%)	20 (17.4%)	0.269
Coronary artery dominance				0.074
Left	92 (10.3%)	32 (13.0%)	8 (7.0%)	
Right	745 (83.1%)	192 (78.0%)	93 (80.9%)	
Balanced	59 (6.6%)	22 (8.9%)	14 (12.2%)	
Stenting technique				0.293
Single	512 (57.1%)	137 (55.7%)	70 (60.9%)	
T-stenting	43 (4.80%)	6 (2.44%)	1 (0.87%)	
Culotte stenting	5 (0.56%)	1 (0.41%)	1 (0.87%)	
Kissing balloon	336 (37.5%)	102 (41.5%)	43 (37.4%)	
Final kissing balloon	389 (43.4%)	112 (45.5%)	37 (32.2%)	0.045

Data are number of patients (%).

**Table 3:** Ten-year clinical outcome.

Outcome	Without Diabetes (*n* = 896)	With Diabetes (*n* = 361)		Hazard Ratio with 95% Confidence Interval		
		On Oral Antidiabetic Drugs (*n* = 246)	On Insulin (*n* = 115)	On Oral Antidiabetic Drugs vs. No Diabetes	On Insulin vs. No Diabetes	On Insulin vs. Oral Antidiabetic Drugs
All-cause death	291 (35.7)	111 (49.5)	75 (70.0)	1.57 [1.26–1.96]	2.80 [2.17–3.61]	1.78 [1.33–2.39]
Cardiac death	181 (22.6)	62 (27.9)	42 (40.1)	1.44 [1.07–1.92]	2.65 [1.90–3.70]	1.85 [1.24–2.74]
Myocardial infarction	54 (6.2)	7 (3.0)	11 (10.0)	0.49 [0.22–1.07]	1.79 [0.93–3.42]	3.68 [1.43–9.46]
Definite stent thrombosis	13 (1.5)	3 (1.2)	2 (1.8)	0.90 [0.26–3.14]	1.41 [0.32–6.22]	1.56 [0.26–9.50]
Coronary artery bypass graft surgery	23 (2.7)	5 (2.2)	2 (1.7)	0.85 [0.32–2.23]	0.81 [0.19–3.44]	0.96 [0.19–4.92]
Repeat PCI	175 (20.6)	51 (21.8)	25 (22.1)	1.15 [0.84–1.57]	1.36 [0.89–2.08]	1.18 [0.73–1.91]
Target lesion revascularization	190 (22.3)	56 (24.0)	27 (23.8)	1.16 [0.86–1.56]	1.34 [0.89–2.01]	1.15 [0.73–1.83]
Stroke	20 (2.3)	7 (2.9)	2 (1.7)	1.36 [0.58–3.19]	0.90 [0.21–3.81]	0.66 [0.14–3.19]
Nontarget lesion revascularization	360 (41.3)	103 (43.4)	54 (47.7)	1.12 [0.90–1.39]	1.39 [1.05–1.84]	1.24 [0.90–1.71]

Data are number of patients with cumulative incidences calculated by Kaplan-Meier method. For outcomes other than all-cause mortality, cumulative incidences were calculated after accounting for competing risk of death. PCI = percutaneous coronary intervention.

**Table 4:** Hazard ratios (with 95% confidence interval) for 10-year clinical outcomes after adjustment in the Cox proportional hazards model.

Outcome	Adjusted Hazard Ratio with 95% Confidence Interval		
	On Oral Antidiabetic Drugs vs. No Diabetes	On Insulin Therapy vs. No Diabetes	On Insulin Therapy vs. Oral Antidiabetic Drugs
All-cause death	1.23 [0.98–1.55] *	2.10 [1.58–2.78]	1.71 [1.25–2.32]
Cardiac death	1.08 [0.79–1.47]	1.76 [1.18–2.62]	1.64 [1.05–2.56]
Myocardial infarction	0.47 [0.21–1.05]	1.43 [0.67–3.05]	3.03 [1.09–8.43]
Definite stent thrombosis	0.91 [0.23–3.53]	1.23 [0.18–8.26]	1.35 [0.18–10.46]
Coronary artery bypass graft surgery	0.91 [0.33–2.50]	0.57 [0.12–2.75]	0.62 [0.12–3.21]
Repeat PCI	1.07 [0.76–1.50]	1.08 [0.65–1.79]	1.01 [0.59–1.73]
Target lesion revascularization	1.08 [0.78–1.48]	1.05 [0.64–1.71]	0.97 [0.58–1.63]
Stroke	1.35 [0.61–2.97]	0.91 [0.21–4.00]	0.67 [0.14–3.18]
Nontarget lesion revascularization	1.14 [0.90–1.43]	1.34 [0.99–1.82] **	1.18 [0.84–1.65]

Data are adjusted hazard ratios with 95% confidence interval obtained from multivariable Cox proportional hazards model. PCI = percutaneous coronary intervention. * *p* = 0.080 for the association between oral antidiabetic therapy with the risk for all-cause mortality. ** *p* = 0.060 for the association between insulin therapy with the risk of nontarget lesion revascularization.

## Data Availability

All data supporting the findings of this study are available within the paper and its Supplementary Information.

## Supplementary Materials

The following supporting information can be downloaded at: https://www.mdpi.com/article/10.3390/jcm14248851/s1, Table S1. Baseline characteristics in patients without and with diabetes mellitus; Table S2. Angiographic and procedural characteristics in patients without and with diabetes mellitus; Table S3. Therapy at discharge; Table S4. Ten-year clinical outcome according to diabetic status; Table S5. Number, type and location of repeat percutaneous coronary interventions; Table S6. Number, type, location and timing of repeat percutaneous coronary interventions; Table S7. Ten-year clinical outcome according to hemoglobin A1c in patients with diabetes; Figure S1: Time-to-event curves of coronary artery bypass surgery (left panel) and repeat percutaneous coronary intervention (PCI).
