# Supplementary material for: Ten-Year Outcomes of Patients with Left Main Coronary Artery Disease and Diabetes Mellitus Treated with Percutaneous Coronary Intervention
Source: J Clin Med. 2025 Dec 14;14(24):8851. doi: 10.3390/jcm14248851 (PMC12734061; doi:10.3390/jcm14248851)
Supplement: Supplementary file 1 [file jcm-14-08851-s001.zip › jcm-3999092-supplementary.pdf]

Supplemental Material

Supplemental Table S1. Baseline characteristics in patients without and with diabetes mellitus

Supplemental Table S2. Angiographic and procedural characteristics in patients without and with diabetes mellitus

Supplemental Table S3. Therapy at discharge

Supplemental Table S4. Ten-year clinical outcome according to diabetic status

Supplemental Table S5. Number, type and location of repeat percutaneous coronary interventions

Supplemental Table S6. Number, type, location and timing of repeat percutaneous coronary interventions

Supplemental Table S7. Ten-year clinical outcome according to hemoglobin A1c in patients with diabetes

Figure S1: Time-to-event curves of coronary artery bypass surgery (left panel) and repeat percutaneous coronary intervention (PCI)

Table S1. Baseline characteristics in patients without and with diabetes mellitus

Characteristic	Without diabetes (n = 896)	With diabetes (n = 361)	P value
Age (years)	69.4 [62.1;76.2]	70.7 [65.4;77.0]	0.004
Women	206 (23.0%)	95 (26.3%)	0.239
Body mass index (kg/m^2)	26.2 [24.2;28.7]	27.8 [25.2;31.0]	<0.001
Arterial hypertension	592 (66.1%)	276 (76.5%)	<0.001
Hypercholesterolemia	662 (73.9%)	274 (75.9%)	0.503
Current smoking	113 (12.6%)	39 (10.8%)	0.427
Extent of coronary artery disease			<0.001
2-vessel disease<br>3-vessel disease	287 (32.0%)<br>609 (68.0%)	72 (19.9%)<br>289 (80.1%)	
Presentation with ACS	324 (36.2%)	154 (42.7%)	0.037
History of myocardial infarction	257 (28.7%)	101 (28.0%)	0.856
History of PCI	448 (50.0%)	187 (51.8%)	0.606
Serum creatinine (mg/dl)	0.95 [0.80;1.10]	1.00 [0.85;1.28]	<0.001
Left ventricular ejection fraction (%)	58.0 [47.8;62.0]	53.0 [40.0;60.0]	<0.001
Comorbidities			
Peripheral artery disease	70 (7.81%)	56 (15.5%)	<0.001
Chronic obstructive pulmonary disease	20 (2.23%)	20 (5.54%)	0.004
Malignancies	151 (16.9%)	58 (16.1%)	0.799
Vessel size, mm	3.70 [3.38;4.04]	3.63 [3.39;3.99]	0.243
SYNTAX-Score	29.0 [21.0;36.0]	30.0 [22.0;39.0]	0.012
2nd generation drug-eluting stents	465 (51.9%)	185 (51.2%)	0.883
Non left main coronary artery lesions	0.00 [0.00;1.00]	0.00 [0.00;1.00]	0.581

Values are median or number of patients (%)

ACS=acute coronary syndrome; PCI=percutaneous coronary intervention; SYNTAX = SYNergy between PCI with TAXUS and Cardiac Surgery

Table S2. Angiographic and procedural characteristics in patients without and with diabetes mellitus

Characteristic	Without diabetes (n = 896)	With diabetes (n = 361)	P value
Lesion location in the left main			0.154
Ostial	94 (10.5%)	51 (14.1%)	
Distal/bifurcation	655 (73.1%)	248 (68.7%)	
Mid-shaft	147 (16.4%)	62 (17.2%)	
Occluded right coronary artery	103 (11.5%)	50 (13.9%)	0.289
Trifurcation morphology	108 (12.1%)	51 (14.1%)	0.364
Coronary artery dominance			0.099
Left	92 (10.3%)	40 (11.1%)	
Right	745 (83.1%)	285 (78.9%)	
Balanced	59 (6.6%)	36 (10.0%)	
Stenting technique			0.115
Single	512 (57.1%)	207 (57.3%)	
T-stenting	43 (4.8%)	7 (1.9%)	
Culotte stenting	5 (0.56%)	2 (0.55%)	
Kissing balloon technique	336 (37.5%)	145 (40.2%)	
Final kissing balloon	389 (43.4%)	149 (41.3%)	0.528

Values are number of patients (%)

Table S3. Therapy at discharge

Drugs	Without diabetes (n= 896)	With diabetes (n=361)		P value
		On oral antidiabetic drugs (n=246)	On insulin therapy (n=115)	
Therapy at discharge				
Statins	835 (93.2%)	227 (92.3%)	102 (88.7%)	0.217
Angiotensin-converting enzyme inhibitors	726 (81.0%)	199 (80.9%)	98 (85.2%)	0.541
Angiotensin II type 1 receptor blockers	128 (14.3%)	31 (12.6%)	18 (15.7%)	0.701
Beta-blockers	856 (95.5%)	229 (93.1%)	110 (95.7%)	0.279

Data are numbers of patients (%)

Table S4. Ten-year clinical outcome according to diabetic status

Outcome	Without diabetes (n=896)	With diabetes (n=361)	Hazard ratio with 95% confidence interval	
			Unadjusted	Adjusted
All-cause death	291 (35.7)	186 (56.1)	1.91 [1.59 - 2.30]	1.46 [1.19 - 1.78]
Cardiac death	181 (22.6)	104 (31.8)	1.76 [1.38 - 2.24]	1.27 [0.97 - 1.65]
Myocardial infarction	54 (6.2)	18 (5.2)	0.88 [0.51 - 1.49]	0.77 [0.44 - 1.36]
Definite stent thrombosis	13 (1.5)	5 (1.4)	1.05 [0.38 - 2.91]	1.01 [0.29 - 3.46]
Coronary artery bypass graft surgery	23 (2.7)	7 (2.0)	0.84 [0.36 - 1.95]	0.79 [0.30 - 2.08]
Repeat PCI	175 (20.6)	76 (21.9)	1.21 [0.93 - 1.59]	1.07 [0.79 - 1.46]
Target lesion revascularization	190 (22.3)	83 (23.9)	1.21 [0.94 - 1.57]	1.09 [0.81 - 1.46]
Stroke	20 (2.3)	9 (2.5)	1.22 [0.56 - 2.65]	1.29 [0.61 - 2.72]
Nontarget lesion revascularization	360 (41.3)	157 (44.8)	1.20 [1.00 - 1.44]	1.20 [0.98 - 1.47]

Data are number of patients with cumulative incidences calculated by Kaplan-Meier method. For outcomes other than all-cause mortality, cumulative incidences were calculated after accounting for competing risk of death. PCI=percutaneous coronary intervention

Table S5. Number, type and location of repeat percutaneous coronary interventions

Characteristic	All procedures (n=321)	Without diabetes (n=222)	With diabetes (n=99)		P value
			On oral drugs (n=62)	On insulin (n=37)	
Number of repeat PCI					0.008
1	205 (63.9%)	146 (65.8%)	43 (69.4%)	16 (43.2%)	
2	58 (18.1%)	34 (15.3%)	12 (19.4%)	12 (32.4%)	
3	33 (10.3%)	21 (9.46%)	3 (4.84%)	9 (24.3%)	
4	20 (6.23%)	16 (7.21%)	4 (6.45%)	0 (0.00%)	
5	5 (1.56%)	5 (2.25%)	0 (0.00%)	0 (0.00%)	
Type of intervention					0.764
Plain balloon angioplasty	165 (51.4%)	115 (51.8%)	33 (53.2%)	17 (45.9%)	
Stenting	156 (48.6%)	107 (48.2%)	29 (46.8%)	20 (54.1%)	
Location of intervention					0.005
LAD	54 (16.8%)	46 (20.7%)	7 (11.3%)	1 (2.70%)	
LCA	176 (54.8%)	123 (55.4%)	29 (46.8%)	24 (64.9%)	
LCx	91 (28.3%)	53 (23.9%)	26 (41.9%)	12 (32.4%)	

Values are number of interventions (%)

LAD = left anterior descending coronary artery; LCA = left coronary artery; LCx = left circumflex coronary artery; PCI = percutaneous coronary intervention

Table S6. Number, type, location and timing of repeat percutaneous coronary interventions

Characteristic	Number of repeat percutaneous coronary interventions						P Value
	All (n=321)	1 (n=205)	2 (n=58)	3 (n=33)	4 (n=20)	5 (n=5)	
Type of intervention							0.058
Plain balloon angioplasty	165 (51.4%)	103 (50.2%)	26 (44.8%)	20 (60.6%)	15 (75.0%)	1 (20.0%)	
Stenting	156 (48.6%)	102 (49.8%)	32 (55.2%)	13 (39.4%)	5 (25.0%)	4 (80.0%)	
Location of intervention							<0.001
LAD	54 (16.8%)	42 (20.5%)	8 (13.8%)	0 (0.00%)	0 (0.0%)	4 (80.0%)	
LCA	176 (54.8%)	101 (49.3%)	31 (53.4%)	29 (87.9%)	15 (75.0%)	0 (0.0%)	
LCx	91 (28.3%)	62 (30.2%)	19 (32.8%)	4 (12.1%)	5 (25.0%)	1 (20.0%)	
Time interval to first repeat PCI (days)	1048±1079	947±1043	1001±1029	1564±1232	1014±1024	2480±437	0.001
Time interval between repeat PCIs (days)	874±980	947±1043	608±814	963±857	595±736	1510±1125	0.050

Values are number of events (%) or mean ± standard deviation

LAD = left anterior descending coronary artery; LCA = left coronary artery; LCx = left circumflex coronary artery; PCI = percutaneous coronary intervention

Table S7. Ten-year clinical outcome according to hemoglobin A1c in patients with diabetes

Outcome	Hemoglobin A1c <6.9% (n=146)	Hemoglobin A1c ≥6.9% (n=146)	Unadjusted Hazard ratio [95% confidence interval]
All-cause death	76 (55.0)	76 (54.2)	0.92 [0.67 - 1.27]
Cardiac death	44 (33.2)	43 (30.7)	0.90 [0.59 - 1.37]
Myocardial infarction	11 (7.7)	3 (2.1)	0.26 [0.07 - 0.91]
Definite stent thrombosis	3 (2.1)	1 (0.7)	0.33 [0.03 - 3.16]
Coronary artery bypass graft surgery	3 (2.1)	1 (0.7)	0.31 [0.03 - 2.90]
Repeat PCI	39 (27.4)	33 (23.0)	0.77 [0.49 - 1.23]
Target lesion revascularization	42 (29.5)	34 (23.7)	0.73 [0.44 - 1.22]
Stroke	5 (3.4)	3 (2.1)	0.59 [0.14 - 2.46]
Nontarget lesion revascularization	71 (49.4)	67 (46.5)	0.84 [0.60 - 1.17]

Data are number of patients with cumulative incidences calculated by Kaplan-Meier method. For outcomes other than all-cause mortality, cumulative incidences were calculated after accounting for competing risk of death. PCI=percutaneous coronary intervention

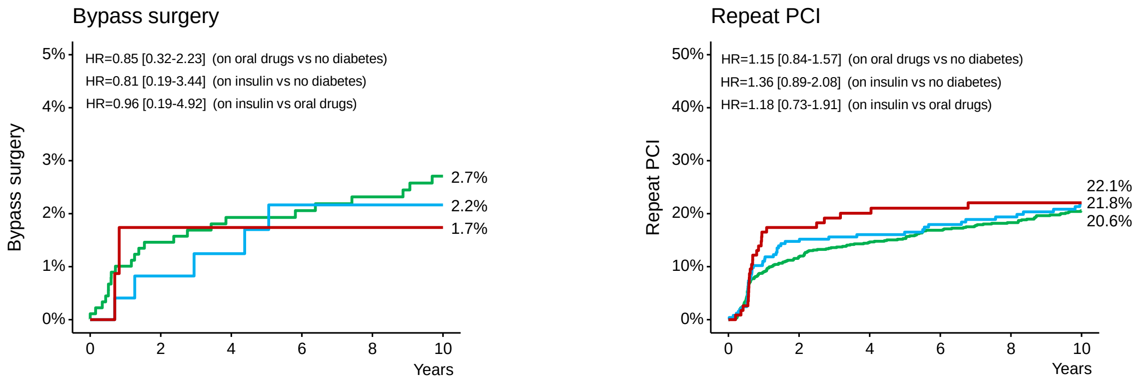

Number at risk

No Diabetes	895	800	687	598	528	461
Diabetes (on oral drugs)	246	209	173	141	119	100
Diabetes (on insulin)	115	88	67	49	35	29

Number at risk

No Diabetes	896	722	600	507	444	373
Diabetes (on oral drugs)	246	180	147	115	99	80
Diabetes (on insulin)	115	72	55	39	26	22

Figure S1. Time-to-event curves of coronary artery bypass surgery (left panel) and repeat percutaneous coronary intervention (PCI); HR=hazard ratio
